# Acute Abdominal Pain Revealing Gallbladder Torsion: A Rare Case

**DOI:** 10.7759/cureus.85557

**Published:** 2025-06-08

**Authors:** Haitam Soussan, Kradi Yassin, Abdelali Guellil, Rachid Jabi, Mohammed Bouziane

**Affiliations:** 1 Department of General Surgery, Faculty of Medicine and Pharmacy, Laboratory of Anatomy, Microsurgery and Surgery Experimental and Medical Simulation (LAMCESM), Mohammed First University, Oujda, MAR; 2 Department of General Surgery, Mohammed VI University Hospital, Oujda, MAR

**Keywords:** acute abdominal surgery, cholecystitis, gallbladder volvulus, general emergency surgery, torsion

## Abstract

We present the case of a 91-year-old woman who was diagnosed with acute gallbladder torsion, complicated by gallbladder necrosis and the development of septicemia. The torsion involved the cystic duct and cystic artery. Gallbladder volvulus is a rare but potentially life-threatening condition that necessitates a high index of clinical suspicion to ensure timely surgical management. Although its exact etiology remains unclear, several theories have been proposed to explain its pathogenesis. Prompt diagnosis and surgical management of gallbladder volvulus is important to avoid the morbidity and mortality of gangrenous cholecystitis and biliary peritonitis in a frail old population of patients.

## Introduction

Acute gallbladder torsion is an exceptionally rare pathology. The first case was reported by Wendel in 1898 [1]. Although the exact incidence of this condition remains uncertain, over 500 cases have been documented in recent years [2]. The clinical importance of gallbladder volvulus stems from its potential to progress quickly to ischemia and necrosis, making prompt surgical intervention essential for favorable outcomes. The etiology of this condition is not fully understood, but several hypotheses have been proposed to explain its occurrence [3].

Acute torsion of the gallbladder is defined as the rotation of the gallbladder around its mesentery, along the axis of the cystic duct and cystic artery [3]. We report the case of a 91-year-old woman who presented with acute gallbladder torsion complicated by necrosis and the onset of septicemia. The aim of this report is to raise awareness of this rare condition, contribute to a better understanding of its true incidence, and assist clinicians in making an early diagnosis to prevent the serious complications and mortality associated with it.

## Case presentation

A 91-year-old woman, with no notable past medical history, presented to the emergency department with diffuse abdominal pain that had begun the previous day. The pain had intensified progressively and was accompanied by episodes of vomiting. On arrival, the patient was afebrile but tachycardic, with a heart rate of 110 beats per minute and stable blood pressure. Physical examination revealed tenderness localized to the right upper quadrant of the abdomen, where a palpable mass was also noted.

Initial laboratory investigations indicated a pronounced inflammatory response, with a white blood cell count of 12.5×10⁹/L (normal range: 4.5-11×10⁹/L) and a C-reactive protein (CRP) level of 79 mg/L (normal range: <5 mg/L). Liver function tests remained within normal limits.

**Figure 1:**
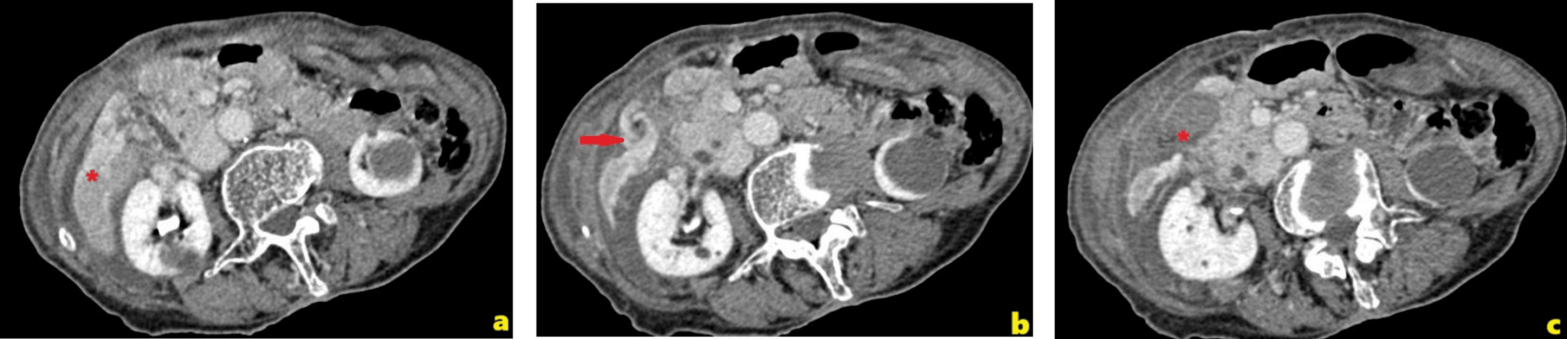
CT scan of the abdomen showing (a) part of the right liver parenchyma demonstrating perfusion disorder, (b) a whirlpool sign of the cystic pedicle, and (c) peritoneal fluid below the whirlpool sign CT: computed tomography

**Figure 2:**
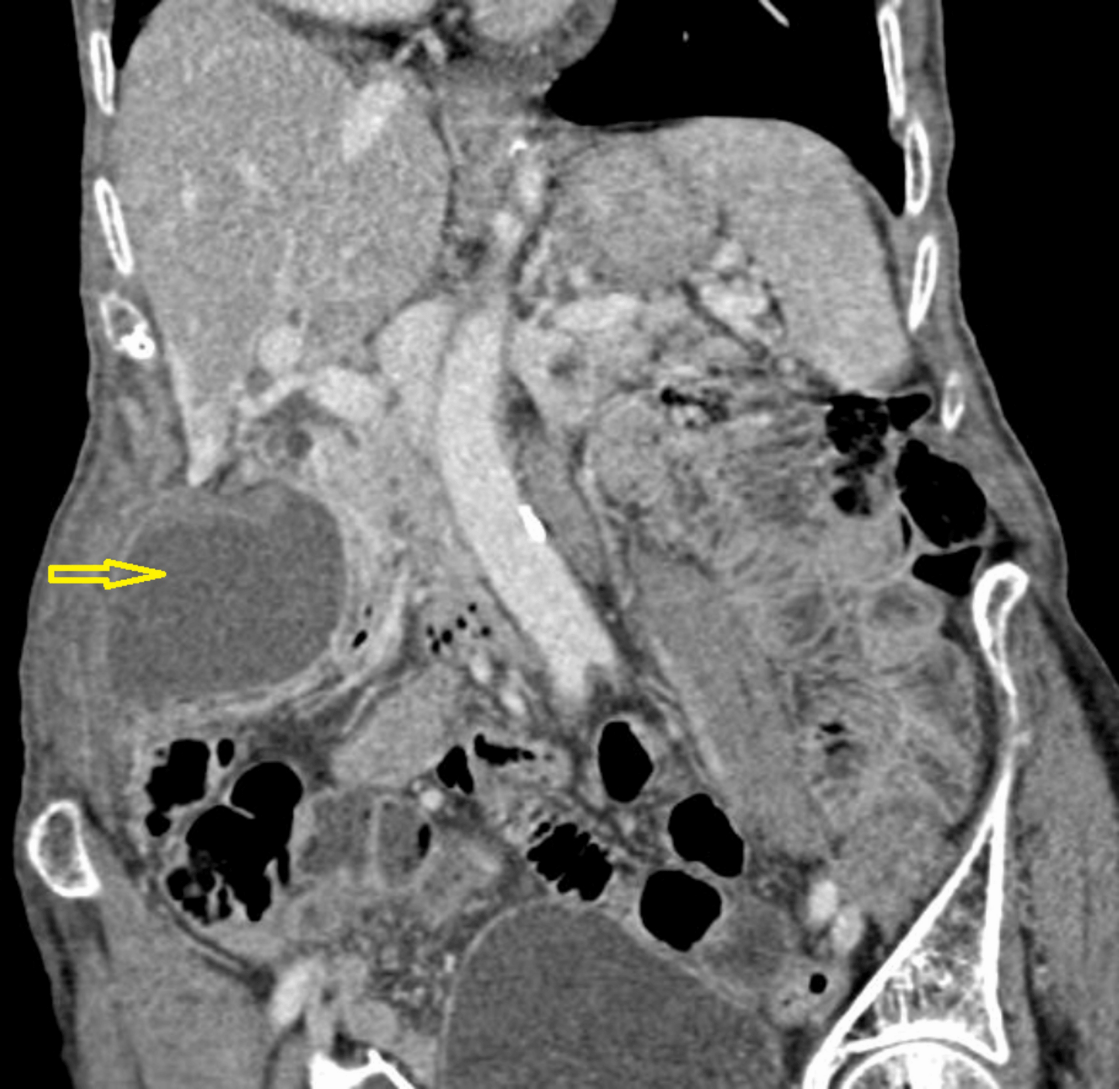
Coronal CT scan showing abnormal arrangement of the distended gallbladder Yellow arrow: distended gallbladder; CT: computed tomography

Given the non-specific clinical presentation and concern for intra-abdominal pathology, a contrast-enhanced abdominal computed tomography (CT) scan was performed. Imaging revealed a markedly distended gallbladder, measuring 9 cm in length, positioned abnormally. The CT also demonstrated areas of wall enhancement loss and perihepatic fluid, along with a stricture at the level of the infundibulum, raising suspicion of a torsion at this site. These findings were highly suggestive of gallbladder volvulus (Figures 1, 2).

**Figure 3:**
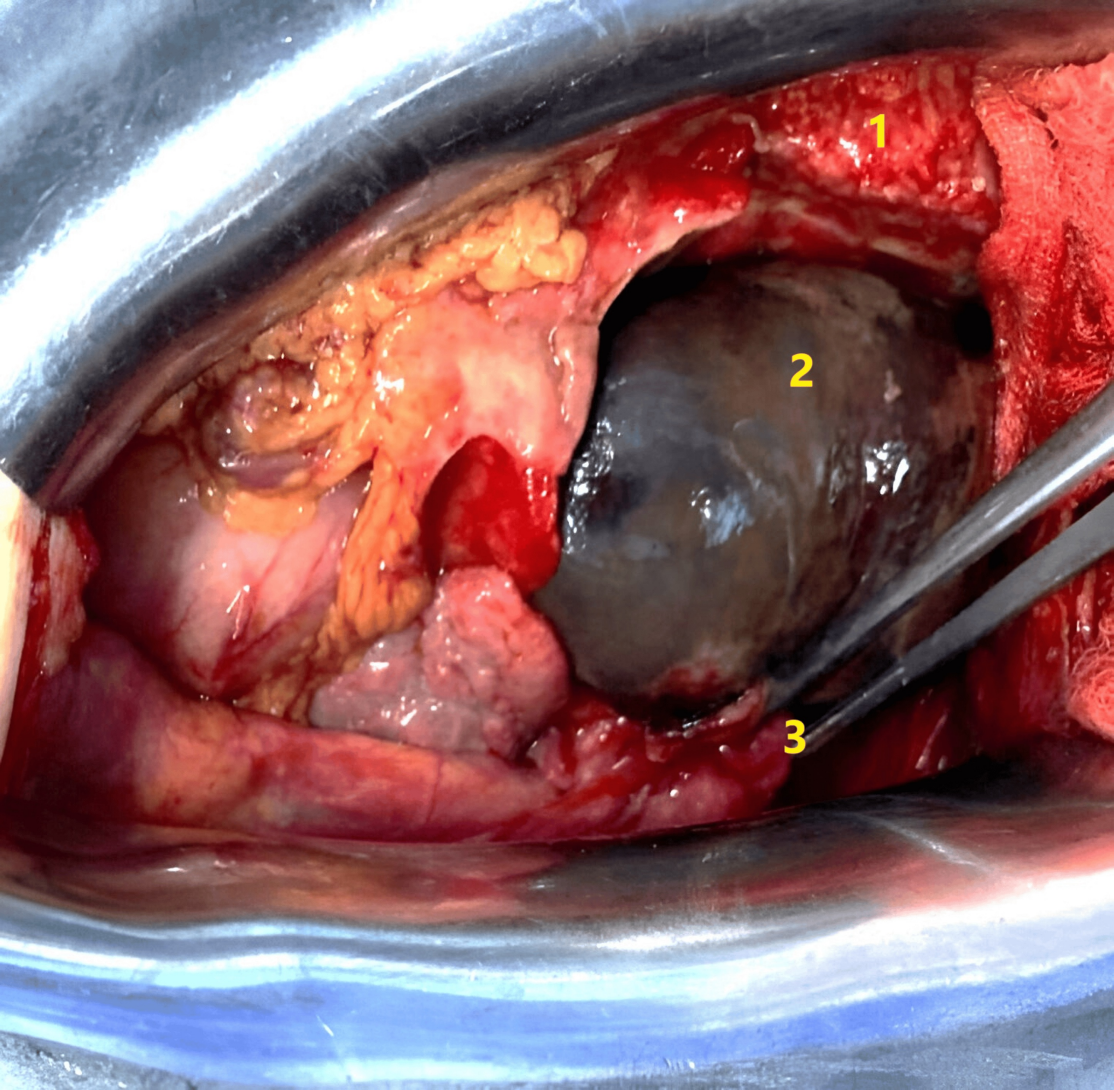
Intraoperative photograph showing gallbladder distension with early signs of necrosis. Upon opening the abdomen, the gallbladder was found twisted around the cystic duct and cystic artery 1: liver; 2: distended and twisted gallbladder; 3: twisted cystic pedicle

**Figure 4:**
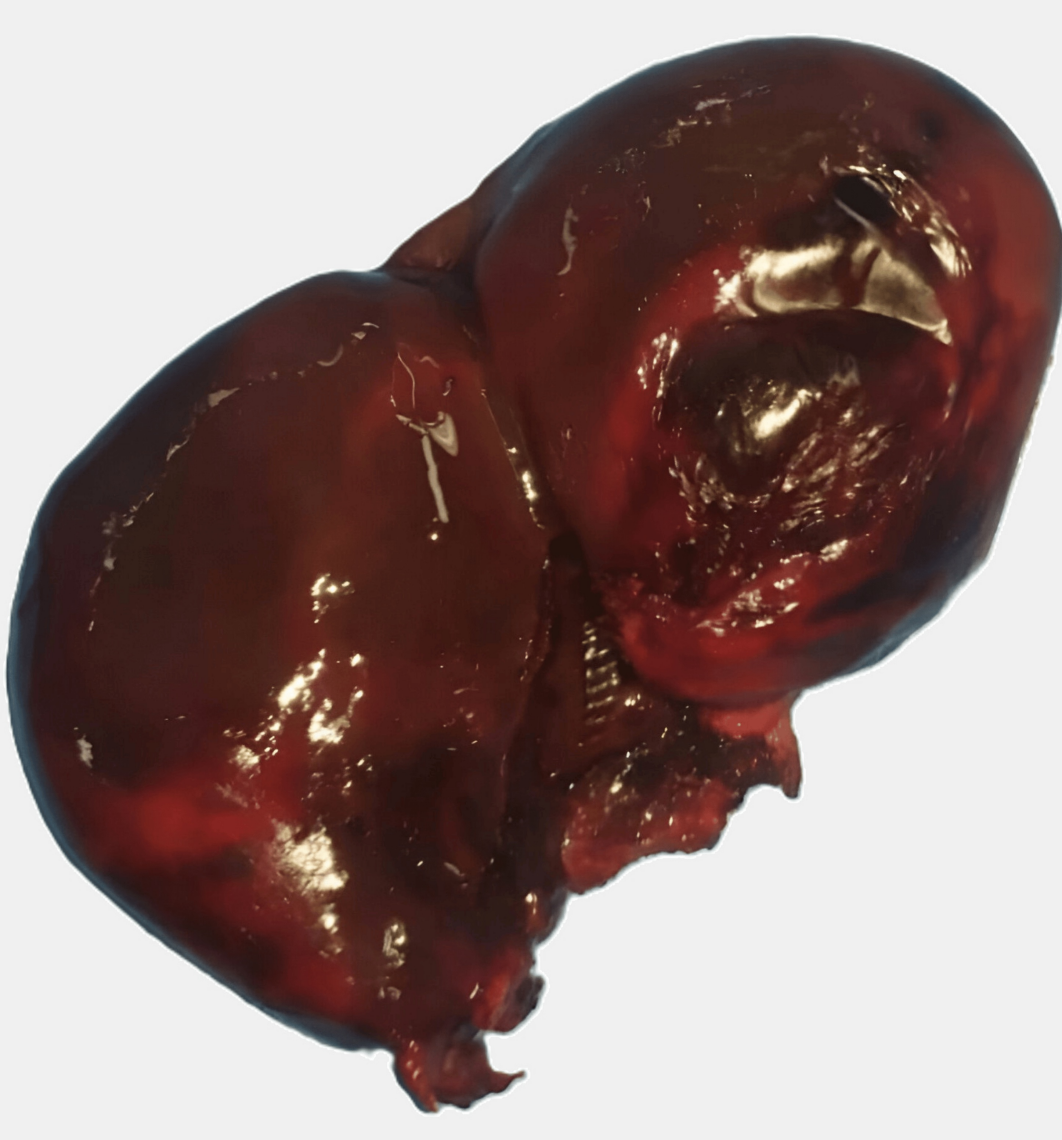
Cholecystectomy specimen showing a necrotic gallbladder wall

The patient underwent emergency surgical exploration. Given the patient's clinical condition and the unavailability of laparoscopic facilities, a laparotomy was performed. Intraoperative findings revealed a markedly distended, completely necrotic gallbladder, with a full 360° clockwise torsion around its mesentery. A cholecystectomy was performed, accompanied by abdominal drainage placed in the subhepatic space (Figures 3, 4). The drain was removed, and the patient was discharged on day 4 without complications. At the one-month follow-up, the postoperative course was uneventful.

The histopathological examination revealed an enlarged gallbladder with the presence of gallstones, an edematous wall, and extensive transmural hemorrhagic necrosis.

## Discussion

Gallbladder volvulus, first described by Wendel in 1898 [1], is a rare pathology, with less than 500 cases reported in the literature [2]. Although it can occur at any age, it is particularly common in the elderly, affecting 84% of people over the age of 54. Additionally, it is more common in women than in men, with a sex ratio of 3:1 [3]. This increased prevalence in women may be associated with the loss of fat and elasticity, which leads to visceroptosis and increased organ mobility [4].

Various causes have been linked to this pathology, one relating to an embryological anomaly in gallbladder migration leading to a long or absent mesocyst [2]. Others relate to visceral fat loss, colonic, duodenal, or gastric peristalsis, and kyphoscoliosis [5].

Gallbladder torsion results in impaired vascularization, rapidly leading to acute ischemia of the organ. This acute ischemia can progress to necrosis, which promotes bacterial proliferation and subsequently increases the risk of severe septicemia, potentially progressing to septic shock, a life-threatening condition [6]. Among other serious and fatal complications associated with this pathology is biliary peritonitis secondary to gallbladder perforation. Mortality associated with this condition can reach 100% in untreated patients, while it is reduced to 3-5% when cholecystectomy is performed in a timely manner [7].

The preoperative diagnosis of gallbladder torsion remains particularly challenging due to the similarity of its symptoms with those of acute calculous cholecystitis. The clinical presentation can vary depending on the degree of torsion; an incomplete torsion (180°) may present with symptoms resembling simple hepatic colic, whereas a complete torsion (360°) can lead to a clinical picture of severe gangrenous cholecystitis [8].

The clinical picture is generally vague, often dominated by acute generalized or even localized right upper quadrant abdominal pain similar to that of acute lithiasis cholecystitis, with or without disturbance of the liver and inflammatory balance [9]. Despite advances in imaging, the diagnosis is often made during surgery, with preoperative diagnosis being made in only 26% of cases [10,11].

Lau et al. have outlined several criteria to assist in the diagnosis of gallbladder torsion. These include patient-related factors such as advanced age, specific morphological characteristics, and the presence of spinal deformities. Clinical features, including the rapid progression of symptoms, early onset of vomiting, and abdominal pain, are also suggestive of this condition. Additionally, physical examination findings, such as the absence of jaundice, the lack of an infectious syndrome, and the absence of tachycardia, may further support the diagnosis [12].

Imaging plays an important role in orienting the diagnosis. Abdominal ultrasound can identify a large "floating vesicle" or vesicular "twist" [5]. CT shows a distended vesicle outside the vesicular bed, with significant parietal edema, the presence of a cystic duct located on the right side of the gallbladder, and the "whirlpool sign" of a twisted cystic artery with medial deviation of the extrahepatic bile duct [13]. Magnetic resonance imaging is the most sensitive examination, but poses the problem of its accessibility in an emergency [5].

The treatment of gallbladder torsion is strictly surgical and must be performed urgently due to the imminent risk of gallbladder gangrene and perforation, which can quickly progress to septicemia and potentially fatal septic shock. While a laparotomy can be employed, laparoscopy is typically preferred [11]. The procedure involves cholecystectomy and abdominal drainage, which is often facilitated by the absence of subhepatic adhesions [9]. Key steps in the surgical intervention include decompression and detorsion of the gallbladder to improve exposure of the anatomical structures. Careful dissection of the cystic artery and duct is critical, as torsion frequently alters the positioning of biliary structures, such as the common bile duct, thereby increasing the risk of biliary injury [13].

## Conclusions

Gallbladder torsion is a rare acute pathology, often confused with acute lithiasis cholecystitis. Although the preoperative diagnosis is difficult to establish, diagnostic orientation can be obtained from clinical examination and radiological explorations. Once gallbladder torsion has been diagnosed, cholecystectomy must be performed urgently to prevent gallbladder gangrene, perforation, and severe peritonitis. Early diagnosis and prompt surgical management ensure a favorable prognosis and prevent serious and potentially fatal complications.
